# Supplementary material for: A genetic program mediates cold-warming response and promotes stress-induced phenoptosis in C. elegans
Source: eLife. 2018 Apr 17;7:e35037. doi: 10.7554/eLife.35037 (PMC5903861; doi:10.7554/eLife.35037)
Supplement: Supplementary file 1. — Shown is a table of fold induction for gene expression levels determined by QPCR measurements of top-ranked randomly selected CW-inducible genes in wild type and zip-10 mutant animals. [file elife-35037-supp1.docx]

**Supplementary File 1:** **Relative mRNA levels of CW-inducible genes in wild-type and *zip-10* mutants.**

Shown is a table of fold induction for gene expression levels determined by QPCR measurements of top-ranked randomly selected CW-inducible genes in wild type and zip-10 mutant animals. WT: wild type; *zip-10* m: *zip-10* mutant (*ok3462*); 1. 25^o^C; 2. 4^o^C-1h, 25^o^C-1h; 3. 4^o^C-2h, 25^o^C-1h; 4. 4^o^C-4h, 25^o^C-1h. N = 3 biological replicates.

| **Gene** |  | **1** | **2** | **3** | **4** | ***P* value** |
| --- | --- | --- | --- | --- | --- | --- |
| *asp-17* | WT | 1.20±0.22 | 9.20±0.07 | 20.38±12.77 | 45.45±12.47 |  |
|  | *zip-10* m | 0.37±0.04 | 0.46±0.12 | 0.71±0.29 | 0.43±0.10 | 0.025 |
| *cpr-3* | WT | 1.01±0.18 | 2.80±0.16 | 3.67±0.10 | 9.75±1.43 |  |
|  | *zip-10* m | 1.24±0.05 | 1.31±0.48 | 1.99±0.40 | 1.30±0.52 | 0.005 |
| *ZK896.4* | WT | 1.01±0.20 | 3.56±1.27 | 6.31±1.13 | 12.22±2.00 |  |
|  | *zip-10* m | 1.19±0.07 | 0.76±0.07 | 1.14±0.07 | 1.03±0.21 | 0.009 |
| *F53A9.1* | WT | 0.80±0.29 | 1.27±0.48 | 2.78±1.07 | 5.51±2.29 |  |
|  | *zip-10* m | 0.69±0.19 | 1.50±0.42 | 2.60±0.62 | 4.95±1.56 | 0.748 |
| *srr-6* | WT | 1.09±0.29 | 4.68±2.84 | 18.31±15.68 | 55.37±33.32 |  |
|  | *zip-10* m | 1.74±0.53 | 37.31±22.32 | 136.49±46.46 | 188.14±64.10 | 0.050 |
| *ceh-37* | WT | 1.04±0.35 | 1.75±0.15 | 2.36±0.56 | 5.52±1.12 |  |
|  | *zip-10* m | 0.76±0.40 | 1.76±0.65 | 2.58±0.57 | 3.53±0.59 | 0.072 |
| *fis-2* | WT | 1.09±0.57 | 0.94±0.36 | 2.92±0.09 | 8.57±2.12 |  |
|  | *zip-10* m | 2.19±0.47 | 3.55±0.44 | 4.57±0.24 | 11.93±1.98 | 0.115 |
| *CYP14A5* | WT | 0.98±0.14 | 2.53±0.46 | 7.54±2.90 | 26.04±5.55 |  |
|  | *zip-10* m | 3.99±1.29 | 6.77±0.77 | 10.12±0.78 | 28.57±5.66 | 0.610 |
| *sqst-1* | WT | 0.98±0.29 | 2.59±1.15 | 4.95±1.08 | 13.59±4.28 |  |
|  | *zip-10* m | 2.68±0.54 | 5.81±0.25 | 8.13±0.59 | 12.85±2.95 | 0.820 |
| *best-5* | WT | 0.97±0.31 | 1.79±0.40 | 5.34±3.24 | 18.21±6.83 |  |
|  | *zip-10* m | 1.37±0.97 | 2.94±0.26 | 6.06±2.23 | 13.35±4.41 | 0.368 |
| *tsp-1* | WT | 1.05±0.41 | 6.78±2.86 | 18.03±6.33 | 72.47±13.72 |  |
|  | *zip-10* m | 3.04±2.34 | 12.53±4.52 | 23.45±2.73 | 50.02±9.17 | 0.108 |
| *dod-3* | WT | 1.08±0.54 | 9.42±4.50 | 18.04±2.08 | 33.69±6.53 |  |
|  | *zip-10* m | 2.64±1.17 | 8.11±2.47 | 13.19±3.75 | 28.77±6.74 | 0.415 |
| *oac-20* | WT | 1.00±0.09 | 4.19±0.56 | 7.92±0.49 | 19.54±3.53 |  |
|  | *zip-10* m | 1.38±0.08 | 2.90±1.17 | 5.05±1.01 | 16.20±4.87 | 0.396 |
| *F37C4.5* | WT | 1.01±0.17 | 2.10±0.38 | 2.79±1.84 | 8.94±2.34 |  |
|  | *zip-10* m | 1.43±0.18 | 1.49±0.27 | 2.19±0.33 | 5.28±1.69 | 0.100 |
| *dod-19* | WT | 1.08±0.45 | 0.95±0.01 | 0.67±0.02 | 1.41±0.37 |  |
|  | *zip-10* m | 1.33±0.07 | 0.85±0.09 | 0.97±0.18 | 1.75±0.23 | 0.262 |
| *dod-22* | WT | 1.15±0.74 | 1.52±0.1. | 1.27±0.22 | 2.76±0.50 |  |
|  | *zip-10* m | 1.41±0.25 | 1.29±0.34 | 2.55±0.24 | 3.41±0.59 | 0.226 |
| *oac-6* | WT | 1.01±0.12 | 3.07±0.77 | 5.36±1.28 | 10.94±3.72 |  |
|  | *zip-10* m | 1.79±0.25 | 2.34±1.30 | 4.80±0.98 | 8.22±1.61 | 0.336 |
| *K09D9.1* | WT | 1.05±0.38 | 1.05±0.58 | 4.01±2.82 | 24.56±3.64 |  |
|  | *zip-10* m | 3.31±1.14 | 7.38±1.61 | 11.55±3.07 | 27.66±8.46 | 0.605 |
| *clec-41* | WT | 1.08±0.49 | 0.83±0.38 | 1.37±0.17 | 2.31±0.54 |  |
|  | *zip-10* m | 1.63±0.27 | 0.35±0.21 | 0.68±0.26 | 1.26±0.19 | 0.065 |
| *F48E3.8* | WT | 1.17±0.72 | 0.87±0.42 | 2.07±0.97 | 5.30±1.14 |  |
|  | *zip-10* m | 0.99±0.27 | 0.60±0.49 | 1.22±0.08 | 5.66±0.66 | 0.666 |
| *cebp-1* | WT | 1.04±0.34 | 1.44±0.22 | 2.84±0.34 | 6.80±3.05 |  |
|  | *zip-10* m | 2.21±0.69 | 2.07±0.37 | 3.01±0.14 | 4.58±0.90 | 0.335 |
